# Hospital Construction

**Published:** 1899-08-19

**Authors:** 


### HOSPITAL CONSTRUCTION.

### THE QUEEN VICTORIA HOSPITAL, MELBOURNE.

The newly-arranged hospital for women, which goes by the name of the Queen Victoria Hospital, was opened by Lady Brassey on July 5th. The speciality of the hospital is that all the members of its acting staff are women. Up to the present out-patients only have been received in the temporary premises which have been in use for the past two or three years, but with the acquisition of the present building there will be room for a dozen in-patients. The institution is a converted house, and there is nothing very particular to be noted about it. Care seems to have been taken to separate the out-patients from the in-patients, as far as this is possible when both are under the same roof. The operating-room is supplied with modern appliances for facilitating asepsis. The walls are of Keen's cement with a dado of porcelain tiles, the basins have treadle taps, the water is passed through a large Berkefeld filter, boiled by a gas heater, and then cooled by being passed in pipes through a cold cistern. If required hot it can be drawn direct from the heater. The floor is of mineral asphalte, which, we think, is rather a good plan. Unless one is very sure of one's foundations cement floors are apt to crack, but asphalte ought to be sufficiently elastic to avoid this danger. Although the members of the acting staff of the new hospital are all women, several of the leading medical men of Melbourne have accepted posts as honorary consultants.

